# Tetrahydrocurcumin Ameliorates Diabetic Cardiomyopathy by Attenuating High Glucose-Induced Oxidative Stress and Fibrosis via Activating the SIRT1 Pathway

**DOI:** 10.1155/2019/6746907

**Published:** 2019-05-09

**Authors:** Kaifeng Li, Mengen Zhai, Liqing Jiang, Fan Song, Bin Zhang, Jie Li, Hua Li, Buying Li, Lin Xia, Lu Xu, Yu Cao, Mengshan He, Hanzhao Zhu, Liyun Zhang, Hongliang Liang, Zhenxiao Jin, Weixun Duan, Siwang Wang

**Affiliations:** ^1^Department of Chinese Materia Medica and Natural Medicines, School of Pharmacy, The Air Force Medical University, Xi'an, 710032 Shaanxi, China; ^2^Department of Cardiovascular Surgery, The First Affiliated Hospital, The Air Force Medical University, Xi'an, 710032 Shaanxi, China; ^3^The 954th Hospital of the People's Liberation Army, Shannan, 850700 Xizang, China; ^4^College of Life Science and Medicine, Northwest University, Xi'an, 710069 Shaanxi, China

## Abstract

Hyperglycemia-induced oxidative stress and fibrosis play a crucial role in the development of diabetic cardiomyopathy (DCM). Tetrahydrocurcumin (THC), a major bioactive metabolite of natural antioxidant curcumin, is reported to exert even more effective antioxidative and superior antifibrotic properties as well as anti-inflammatory and antidiabetic abilities. This study was designed to investigate the potential protective effects of THC on experimental DCM and its underlying mechanisms, pointing to the role of high glucose-induced oxidative stress and interrelated fibrosis. In STZ-induced diabetic mice, oral administration of THC (120 mg/kg/d) for 12 weeks significantly improved the cardiac function and ameliorated myocardial fibrosis and cardiac hypertrophy, accompanied by reduced reactive oxygen species (ROS) generation. Mechanically, THC administration remarkably increased the expression of the SIRT1 signaling pathway both *in vitro* and *in vivo*, further evidenced by decreased downstream molecule Ac-SOD2 and enhanced deacetylated production SOD2, which finally strengthened antioxidative stress capacity proven by repaired activities of SOD and GSH-Px and reduced MDA production. Additionally, THC treatment accomplished its antifibrotic effect by depressing the ROS-induced TGF*β*1/Smad3 signaling pathway followed by reduced expression of cardiac fibrotic markers *α*-SMA, collagen I, and collagen III. Collectively, these finds demonstrated the therapeutic potential of THC treatment to alleviate DCM mainly by attenuating hyperglycemia-induced oxidative stress and fibrosis via activating the SIRT1 pathway.

## 1. Introduction

Diabetic cardiomyopathy (DCM) is a leading cause responsible for the greater risk of morbidity and mortality in diabetic patients, which is currently without available specific treatment in the clinical practice [1, 2]. DCM is characterized by a series of cardiac dysfunction and pathological structural changes including left ventricular dysfunction, cardiac fibrosis, myocardial hypertrophy, and cardiomyocyte apoptosis, in the absence of coronary artery diseases, systemic hypertension, and other heart diseases [3–5]. Multiple mechanisms have been reported to contribute to the pathogenesis of DCM, including unbalanced energy metabolism, oxidative stress, fibrosis, inflammation, and mitochondrial dysfunction [6–8].

Oxidative stress and fibrosis play crucial roles in the occurrence and development of pathological structure and function changes in DCM [6, 7, 9]. Under the conditions of DCM, the antioxidant factors, such as superoxide dismutase (SOD) and glutathione peroxidase (GSH-Px), are sharply declined in heart tissue, and reactive oxygen species (ROS) generation is dramatically increased, which are responsible for cellular oxidative stress [6, 10, 11]. Moreover, continuous stimulation of ROS contributes to activation of the TGF*β*1/Smad3 signaling pathway, which promotes the expression of several pivotal fibrotic markers, such as *α*-SMA, collagen I, and collagen III in hearts of DCM [9, 12]. Thus, therapeutic molecules and agents that target intracellular ROS to further alleviate oxidative stress and related cardiac fibrosis may represent as a potential strategy for DCM.

Tetrahydrocurcumin (THC) is a major bioactive metabolite of the widely studied natural product curcumin, which has been reported to possess a variety of biological properties even superior to curcumin, such as antioxidative, anti-inflammatory, antidiabetic, and neuroprotective effects [13–16]. Murugan and Pari found that THC administration was more effective than curcumin to markedly reduce blood glucose levels and increase the activities of SOD, GSH-Px, and catalase in the livers and kidneys of diabetic rats [17]. It was also reported that THC was found to be more active in decreasing deposition and cross-linking of collagen than curcumin in rats with type 2 diabetes [18]. All those finds revealed the promising potency of THC treatment to prevent DCM in diabetes, which have not been clarified until now.

Sirtuin1 (SIRT1), a cellular nicotinamide adenine dinucleotide- (NAD^+^-) dependent deacetylase, is one of the yeast silent information regulator 2 (Sir2), which plays multiple roles in the cellular biological process such as longevity, senescence, proliferation, DNA repair, apoptosis, inflammation, and cell metabolism [19–21]. SIRT1 is the first member to be discovered in the seven mammalian homolog Sir2 termed sirtuins (SIRT1-SIRT7) and is still the most studied one, especially as a potential target to treat cardiovascular diseases [22]. Recently, we have determined the critical role of SIRT1 in mediating protective effect of natural products to reduce damages in both simple myocardial ischemia/reperfusion and ischemia/reperfusion with type 1 diabetes by attenuating oxidative stress and apoptosis [23, 24]. Of interest, THC was reported to inhibit oxidative stress and extend the life span of Drosophila in a Sir2-dependent way [25]. However, whether THC could attenuate oxidative stress via modulating SIRT1 to prevent the cardiac dysfunction and pathologic structural changes in DCM remains unknown. Therefore, this study was designed to elucidate the potential effects and underlying mechanism of THC treatment to protect against DCM both *in vivo* and *in vitro*.

## 2. Materials and Methods

### 2.1. Animals and Treatments

All animal study procedures were performed in accordance with the Guide for the Care and Use of Laboratory Animals by the National Academy of Sciences and published by the National Institutes of Health (NIH publication no. 86-23, revised 1996). All experimental protocols were reviewed and approved by the Air Force Medical University Committee on Animal Care. Male C57BL/6 mice weighing 20-25 g at the age of 8 weeks were obtained from the Experimental Animal Center of the Air Force Medical University. After fed with high-fat diet (HFD) for 4 weeks, diabetes was established by intraperitoneal injection of streptozotocin (STZ; 60 mg/kg in citrate buffer, pH = 4.5) for three consecutive days, while control mice received citrate buffer of the same volume. The fasting blood glucose (FBG) was monitored on day 7 and day 14 after final STZ injection with a glucometer. Two weeks after STZ injection, mice with FBG ≥ 11.1 mM were considered diabetic. Diabetic mice were randomly assigned into two groups: diabetic cardiomyopathy group (DCM; *n* = 15) and THC-treated DCM group (DCM+THC; *n* = 15). In the DCM+THC group, mice were administrated with THC at a total amount of 120 mg/kg/d once every 2 days for 12 weeks. The DCM group and the age-matched control group (Con; *n* = 15) received the same volume of vehicle alone in the same schedule as the DCM+THC group. Animals were housed under a 12/12 h light/dark cycle at maintained 22°C room temperature. All animals had free access to water, and diabetic mice were fed with HFD while mice in the Con group were fed with normal diet.

### 2.2. Chemicals and Reagents

THC was purchased from Shanghai Macklin Biochemical Co. Ltd. (Shanghai, China) with a purity of 98% tested by high-performance liquid chromatography (HPLC). STZ was purchased from Sigma-Aldrich (St. Louis, MO, USA). The fluorescent probe 2′,7′-dichlorofluorescein diacetate (DCFH-DA) used to detect intracellular ROS generation was purchased from the Beyotime Institute of Biotechnology (Shanghai, China). Dihydroethidium (DHE; for detecting ROS generation in cardiac tissues) were purchased from Invitrogen (Carlsbad, CA, USA). A primary antibody against SOD2 (sc-30080) was obtained from Santa Cruz Biotechnology (CA, USA). Primary antibodies against T-Smad3 (SC101154), p-Smad3 (8760), and *β*-actin (4970) were all purchased from Cell Signaling Technology (Boston, MA, USA). Primary antibodies against SIRT1 (ab110304), acetylated SOD2 (Ac-SOD2, ab137037), *α*-SMA (ab5694), TGF*β*1 (ab92486), collagen І (ab90395), and collagen III (ab7778) were purchased from Abcam (Cambridge, MA, USA). The rabbit anti-goat and goat anti-mouse secondary antibodies were obtained from the Zhongshan Company (Beijing, China).

### 2.3. Cell Culture and Treatments

The H9c2 cardiomyocyte lines used *in vitro* were purchased from Tiancheng Technology (Shanghai, China) and were cultured in Dulbecco's modified Eagle's medium (DMEM; HyClone, Logan, UT, USA) supplemented with 10% (*v*/*v*) fetal bovine serum (FBS; Gibco, Grand Island, NY, USA) and 1% (*v*/*v*) penicillin/streptomycin (Sigma, St. Louis, MO, USA) at 37°C in a humidified atmosphere (95% air and 5% CO_2_). High glucose- (HG-) cultured conditions were used to mimic in vivo DCM. In the HG-treated group, cells were incubated with DMEM containing 33 mmol/L D-glucose, while cells in the THC-treated group were cultured with additional 5 *μ*M THC for 24 h. Cells were incubated in DMEM containing 5.6 mmol/L D-glucose in the low glucose (LG) group as the normal control.

### 2.4. Cell Viability

Cell viability was evaluated using cell counting kit-8 (CCK-8; C008-3#) purchased from 7Sea Pharmatech Co. Ltd. (Shanghai, China) according to the manufacturer's instructions and presented with fold of the optical density for different groups over that of the LG group. Briefly, H9c2 cells were seeded in 96-well plastic plates at a density of 8 × 10^3^ cells/well and cultured with LG, HG, or additional THC treatment as mentioned above. 10 *μ*L CCK-8 solution was added to each well and incubated darkly for 2 h at 37°C before measurement. Absorbance at 450 nm was detected spectrophotometrically by a SpectraMax M5 instrument (Molecular Devices, CA, USA).

### 2.5. Echocardiography

Mouse cardiac function was evaluated by transthoracic echocardiography performed with a VisualSonics Vevo 770 ultrasound system (Toronto, Ontario, Canada). Animals were anesthetized with maintaining heart rates at 400-500 beats per minute by adjusted inhalant 1.0% isoflurane in oxygen and were placed on a warming pad during the whole process. A 30 MHz linear transducer was used to measure left ventricular end-diastolic diameter (LVEDd), left ventricular end-systolic diameter (LVESd), left ventricle internal dimension in diastole (LVIDd) and systole (LVIDs), interventricular septal thickness in diastole (IVSd) and systole (IVSs), and left ventricular posterior wall thickness at end diastole (LVPWd) and systole (LVPWs) at the level of papillary muscles under M mode. Left ventricular ejection fraction (LVEF), left ventricular fractional shortening (LVFS), left ventricular end-systolic volumes (LVESV), and left ventricular end-diastolic volumes (LVEDV) were calculated by computer algorithms. All measurements were based on 6 consecutive cardiac cycles and performed by a blinded investigator.

### 2.6. ROS Detection

Intracellular ROS levels were detected by DCFH-DA in H9c2 cells and by DHE in myocardial frozen sections as previously described [24, 26]. Treated H9c2 cells or tissue section was examined with an Olympus FV1000 (Olympus, Tokyo, Japan) laser confocal microscope after staining by suitable oxidative fluorescent dye according to the manufacturer's instruction. The mean intensity of fluorescence was determined using Image-Pro 6.0 (Media Cybernetics, Bethesda, MD, USA).

### 2.7. Determination of SOD, GSH-Px, and MDA

The activity for superoxide dismutase (SOD) and glutathione peroxidase (GSH-Px) and content of malondialdehyde (MDA) in heart tissues were determined using commercially available SOD (A001-3#), GSH-Px (A005-1#), and MDA (A003-1#) kits obtained from the Institute of Nanjing Jiancheng Bioengineering Institute (Nanjing, Jiangsu, China), following the instructions of the manufacturer. The data were analyzed spectrophotometrically with a SpectraMax M5 instrument (Molecular Devices, CA, USA).

### 2.8. TUNEL Assay

Terminal deoxynucleotidyl transferase dUTP nick end labeling (TUNEL) staining was performed to detect the DNA fragmentation of apoptotic cells by using the In Situ Cell Death Detection kit (Roche Molecular Biochemicals, Mannheim, Germany) following the manufacturer's instructions. The nucleus was visualized by DAPI (4′,6′-diamidino-2-phenylindole) staining after the TUNEL reaction. Images were obtained using an Olympus FV1000 (Olympus, Tokyo, Japan) laser confocal microscope, in which TUNEL-positive cells produced green fluorescence and DAPI displayed blue fluorescence. The apoptotic ratio was expressed as the percentage of the TUNEL-positive apoptotic myocytes to the total number of myocytes.

### 2.9. Histological and Immunohistochemistry Staining

Mouse hearts fixed in 4% paraformaldehyde solution were embedded in paraffin and sectioned at 5 *μ*m. Sections were stained with hematoxylin-eosin (H-E) staining to observe the morphology of cardiac tissue and Masson's trichrome staining to examine extracellular collagen deposition. Immunohistochemistry staining was used to further detect the accumulation of collagen І and collagen III, respectively. After performing antigen retrieval with citrate (pH = 6) and blocking nonspecific binding sites by 8% goat serum, sections were incubated with primary antibody collagen І (1 : 50) or collagen III (1 : 50) at 4°C overnight. Sections were then incubated with anti-mouse or rabbit secondary antibodies labeled with horse radish peroxidase after being washed with PBS for 3 min × 5 times. Staining was achieved by DAB after being washed with PBS and counterstained with hematoxylin.

### 2.10. Transmission Electron Microscopy (TEM)

TEM was performed to observe ultrastructure morphology of myofibril and mitochondrion in myocardium as previously described [21]. Briefly, ultrathin sections with a thickness of 60-64 nm were cut from heart tissue of the left ventricular myocardium. After fixation, sections were performed with alcohol dehydration, embedding, polymerization, sectioning, and staining. Images were obtained with an electron microscope (JEM-2000EX TEM, JEOL Ltd., Tokyo, Japan).

### 2.11. Western Blot

Total protein of the cell and heart tissue were isolated and prepared as previously described [27]. After quantifying with bicinchoninic acid (BCA), the proteins were separated by sodium dodecyl sulfate-polyacrylamide gel electrophoresis (SDS-PAGE) and transferred to a polyvinylidene fluoride (PVDF) membrane. After blocking in TBST buffer (150 mM NaCl, 50 mM Tris, pH 7.5, with 0.1% Tween 20) containing 5% nonfat milk at room temperature for 2 h, the membranes were incubated with primary antibodies against SIRT1 (1 : 1,000), Ac-SOD2 (1 : 1,000), SOD2 (1 : 1,000), TGF*β*1 (1 : 1,000), p-Smad3 (1 : 1000), T-Smad3 (1 : 1,000), *α*-SMA (1 : 2,000), and *β*-actin (1 : 5,000) overnight at 4°C. Then, an appropriate secondary horseradish peroxidase- (HRP-) conjugated antibody (1 : 5,000) was incubated with the membranes for 2 h at room temperature. Image Lab 5.2.1 (Bio-Rad Laboratories, CA, USA) was used to analyze the blots after detection using enhanced chemiluminescence (Millipore).

### 2.12. Statistical Analysis

Data was presented as mean ± standard error of the mean (SEM). The statistical significance of differences between groups was evaluated by the one-way analysis of variance (ANOVA) test followed by the post hoc Tukey test in Prism 5.0 (GraphPad Software, San Diego, CA, USA). A *P* value < 0.05 (two-tailed) was considered statistically significant.

## 3. Results

### 3.1. THC Prevented HG-Induced Cardiomyocyte Death in H9c2 Cells

**Figure 1:**
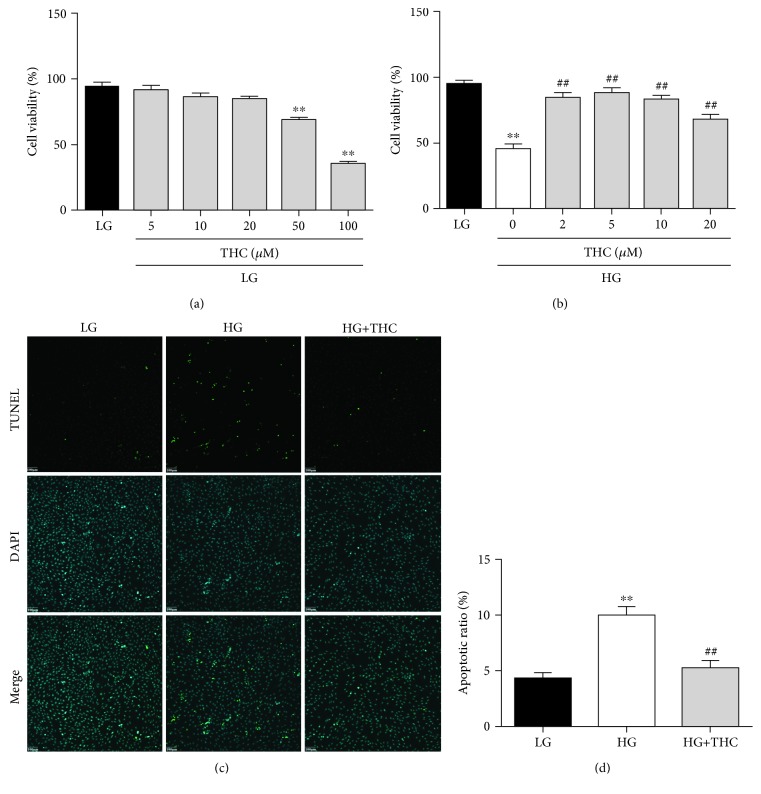
THC prevented HG-induced cardiomyocyte death in H9c2 cells. (a) THC treatment with concentration under 20 *μ*M showed no significant effect on the cell viability of normal cultured H9c2 cells. (b) THC treatment markedly increased the cell viability in HG-induced H9c2 cells with the most effective dose of 5 *μ*M. (c) Representative images of TUNEL staining in H9c2 cells (scale bar = 100 *μ*m). (d) Apoptotic ratio calculated from the proportion of TUNEL-positive nuclei. Data are presented as mean ± SEM. ^∗^^/^^∗∗^*P* < 0.05/0.01 versus the LG group; ^#/##^*P* < 0.05/0.01 versus the HG group.

To investigate the positive effect of THC on preventing cardiomyocytes against high glucose-induced damage and cell death, H9c2 cells were used to be treated with THC at different concentrations when incubated with either low glucose (LG; 5.6 mmol/L) or high glucose (HG; 33 mmol/L) for 24 h, and then, cell viability was detected by CCK-8. As shown in Figure 1(a), THC treatment had no toxic effect on normally cultured cell survival with LG under the concentration of 20 *μ*M, while it could significantly increase cell viability in HG-cultured H9c2 cells (Figure 1(b)). The protective effect was most obvious at a THC concentration of 5 *μ*M (Figure 1(b)), which was chosen for further studies. The protective effectiveness of THC against HG-induced cardiomyocyte death was further determined by TUNEL staining (Figure 1(c)) with significantly decreased TUNEL-positive nuclei and apoptotic ratio (Figure 1(d)) at the THC-treated HG group compared with the HG group.

### 3.2. THC Blocked HG-Induced ROS Generation via Enhancing the SIRT1 Antioxidative Pathway *In Vitro*

To explore the antioxidative effect of THC treatment *in vitro*, DCFH-DA fluorescence was used to detect ROS generation in H9c2 cells. As shown in Figures 1(a) and 1(b), after being cultured with HG for 24 h, ROS generation was dramatically increased in H9c2 cells compared to the LG-cultured group. Intriguingly, THC treatment with the concentration of 5 *μ*M significantly reduced HG-triggered intracellular ROS production in the HG+THC group.

**Figure 2:**
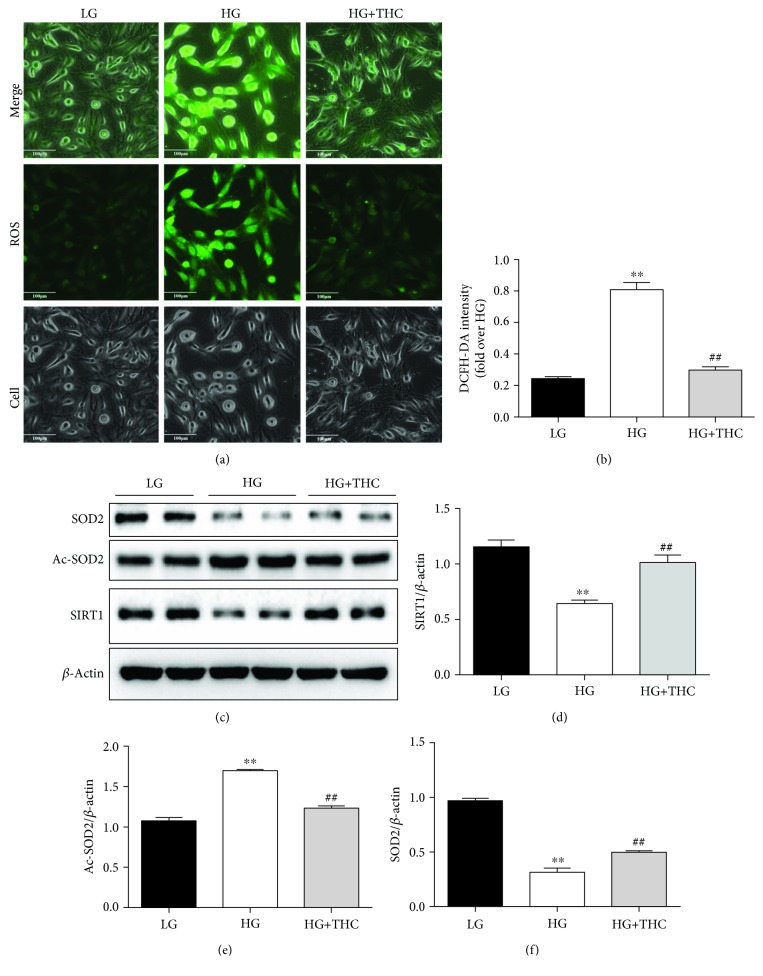
THC reduces high glucose-induced ROS via activating the SIRT1 pathway in H9c2 cells. (a) Representative images of DCFH-DA staining (scale bar = 100 *μ*m). (b) DCFH-DA intensity. (c) Representative blots of SIRT1, Ac-SOD2, SOD2, and *β*-actin. (d–f) Histogram shows the quantitative expression changes of SIRT1, Ac-SOD2, and SOD2; all of the proteins were normalized to *β*-actin before relative quantitative analysis, and all experiments were repeated 4 times independently. Data are presented as mean ± SEM. ^∗^^/^^∗∗^*P* < 0.05/0.01 versus the LG group; ^#/##^*P* < 0.05/0.01 versus the HG group.

We further examined the expression of SIRT1 and relative antioxidative pathways. As Western blot results show in Figures 2(c) and 2(d), SIRT1 expression significantly decreased in the HG-treated group compared with the LG-cultured group. SOD2, a vital molecule in maintaining ROS homeostasis, is also a crucial production of SIRT1 by deacetylating Ac-SOD2. As results show in Figures 2(e) and 2(f), we detected significant reduction of SOD2 accompanied by a remarkable increase of Ac-SOD2 in the HG group. However, treatment with THC observably restored those changes in the HG+THC group. Those results indicate the antioxidative capacity of THC to reduce ROS generation via enhancing the SIRT1 signal pathway.

### 3.3. THC Ameliorates Diabetes-Induced Hypertrophy and Myocardial Dysfunction *In Vivo*

**Figure 3:**
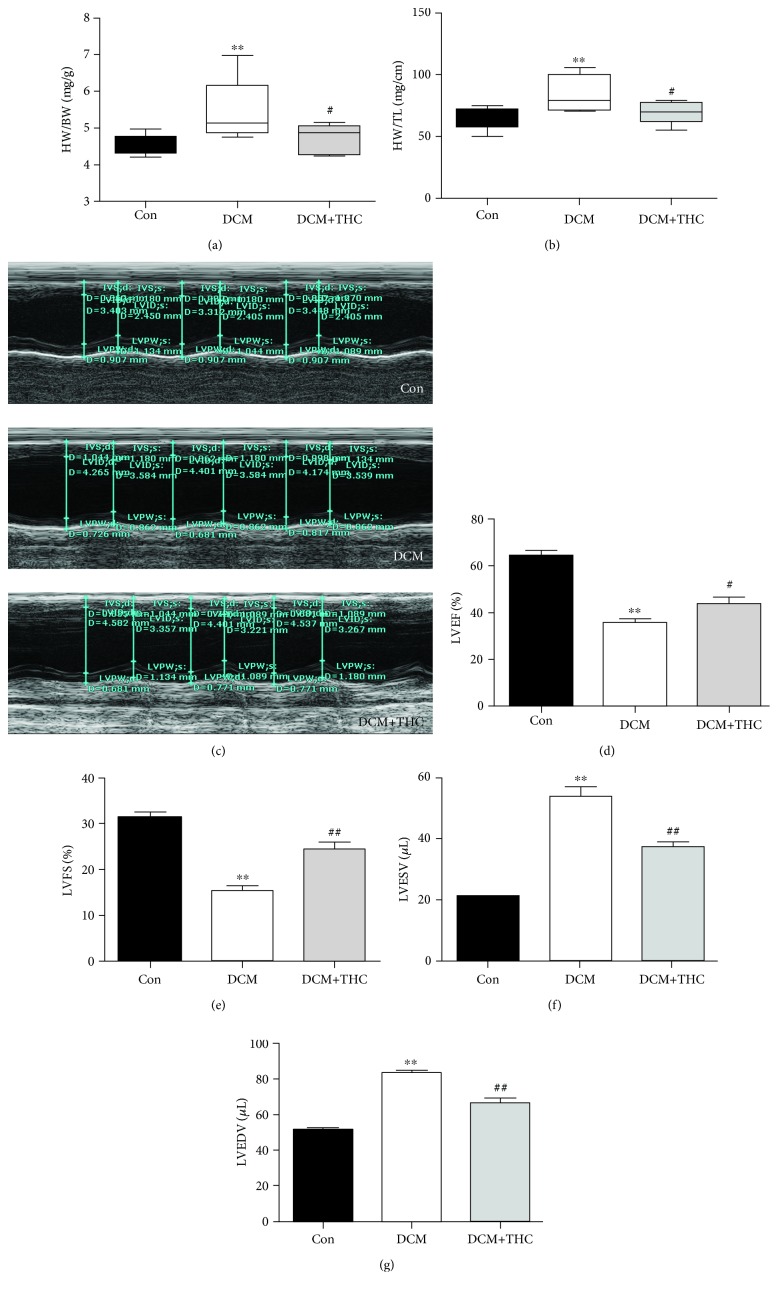
THC prevents cardiac hypertrophy and dysfunction in diabetic mouse: (a) heart weight/body weight (HW/BW) ratio; (b) heart weight/tibia length (HW/TL) ratio; (c) representative images of M-mode echocardiography; (d) left ventricular ejection fraction (LVEF); (e) left ventricular fraction shortening (LVFS); (f) left ventricular end-systolic volume (LVESV); (g) left ventricular end-diastolic volume (LVEDV). Data are presented as the mean ± SEM (*n* = 6 in each group). ^∗^^/^^∗∗^*P* < 0.05/0.01 versus the Con group; ^#/##^*P* < 0.05/0.01 versus the DCM group.

Ventricular hypertrophy and impaired cardiac systolic and diastolic dysfunction are prominent features of DCM hearts. As shown in Figures 3(a) and 3(b), cardiac hypertrophy in DCM mice was evidenced by the increased heart weight/body weight (HW/BW) ratio and enhanced heart weight/tibia length (HW/TL) ratio. Results of echocardiography to assess cardiac functions are shown in Figures 3(c)–3(e). Two major indicators of heart function, left ventricular ejection fraction (LVEF) and left ventricular fractional shortening (LVFS), were markedly decreased in DCM mice. Comparing with DCM mice, THC treatment (120 mg/kg/d for 12 weeks) significantly improved LVEF and LVFS with a dramatically decreased HW/BW and HW/TL ratio in the DCM+THC mouse heart. Furthermore, both left ventricular end-systolic volumes (LVESV) and left ventricular end-diastolic volumes (LVEDV) were markedly increased in DCM mice as compared with Con mice, while THC significantly reduced LVESV and LVEDV in DCM+THC mice (Figures 3(f) and 3(g)). All those data confirmed the cardioprotective effects by THC administration to ameliorate myocardial hypertrophy and dysfunction.

### 3.4. THC Treatment Mitigated Fibrosis and Pathogenic Structure Disorder in Diabetic Myocardium

**Figure 4:**
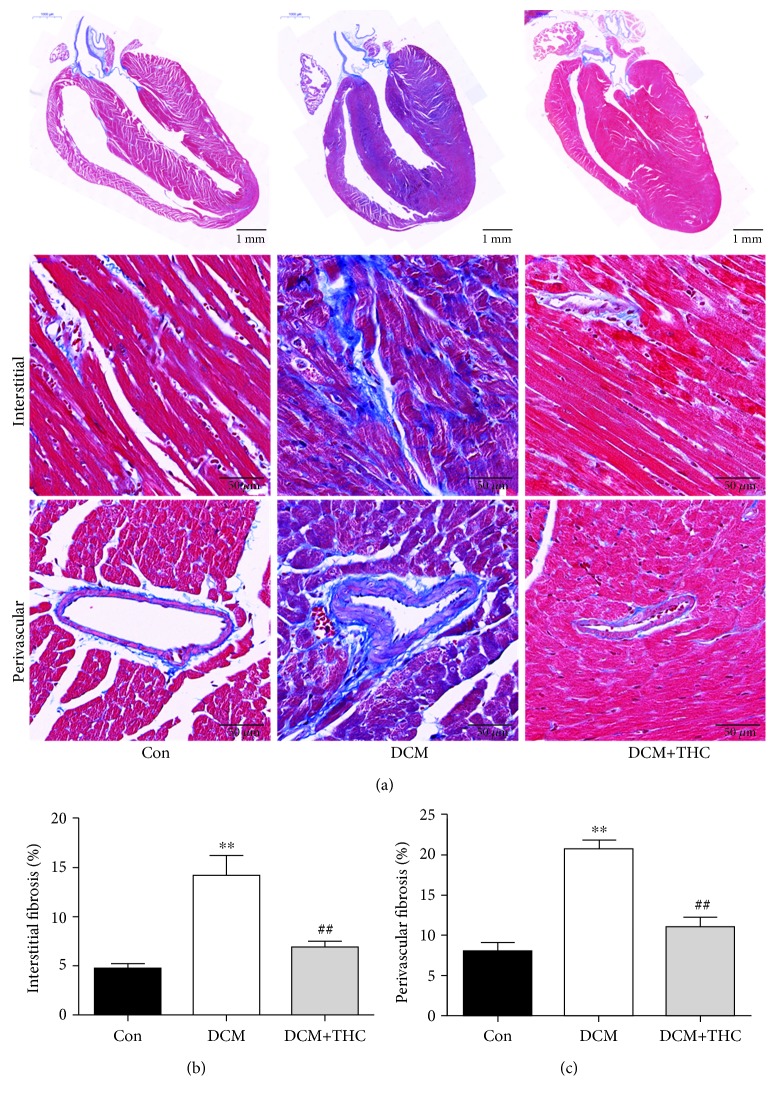
THC alleviated pathogenic fibrosis in interstitial and perivascular areas of diabetic cardiomyopathy. (a) Representative Masson's trichrome staining of the cardiac longitudinal section (scale bar = 1 mm) and interstitial (scale bar = 50 *μ*m) and perivascular (scale bar = 50 *μ*m) areas. (b) Mean cardiac interstitial fibrosis in the heart tissues. (c) Mean cardiac perivascular fibrosis in the heart tissues. Data are presented as the mean ± SEM (*n* = 6 in each group). ^∗^^/^^∗∗^*P* < 0.05/0.01 versus the Con group; ^#/##^*P* < 0.05/0.01 versus the DCM group.

**Figure 5:**
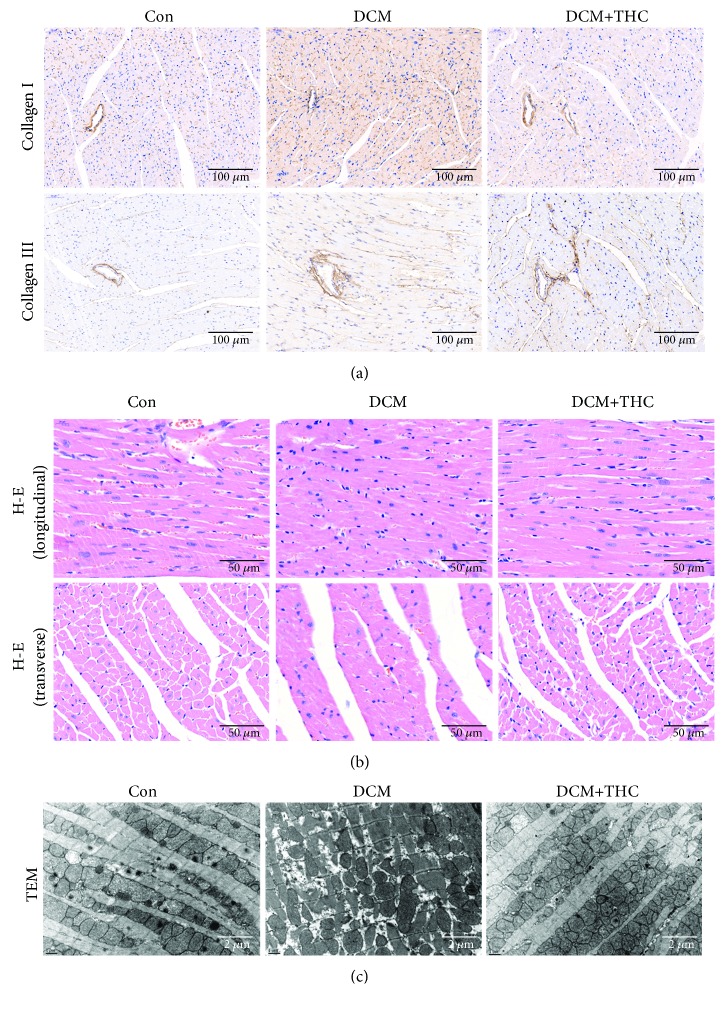
THC relieved collagen deposition and pathogenic structure disorder in diabetic cardiomyopathy. (a) Representative images of immunohistochemistry for collagen І/III. (b) Representative section of hematoxylin and eosin (H-E) staining for myocardial tissue both in longitudinal and transverse sections. (c) Representative transmission electron micrographs of cardiac tissues from left ventricles.

Cardiac fibrosis plays a critical role in the pathogenic structure changes and remodeling of diabetic hearts, which further contributes to myocardial dysfunction in DCM. To gain insight into the effect of THC treatment on cardiac fibrosis, Masson staining was used to detect the levels of collagen deposition. Representative images of Masson staining for the whole cardiac longitudinal section and interstitial and perivascular areas are shown in Figure 4(a). Compared to the Con group, DCM mouse hearts displayed markedly increased collagen content both in interstitial and perivascular areas. THC administration prominently mitigated collagen accumulation of all regions in DCM heart tissues (Figures 4(b) and 4(c)). We further determined the alleviative effect of THC treatment on cardiac fibrosis by detecting collagen І and collagen III deposition through immunohistochemical staining, which show a similar trend consistent with Masson staining (Figure 5(a)).

As shown in Figure 5(b), representative images of H-E staining from both longitudinal and transverse sections displayed grievous pathogenic structural changes and remodeling in the DCM mouse hearts compared to the Con group, especially characterized by disordered cellular structures of cardiomyocytes and hypertrophic myocardium. Markedly, with the administration of THC, a majority of those pathologic characteristics were alleviated in the mouse heart of the DCM+THC group. The representation of transmission electron micrographs (TEM) further verified the protective effect of THC in a more microscopic vision (Figure 5(c)). Cardiomyocytes from mice hearts of the Con group displayed a normal myocardial structure, in which regular sarcomeres composed continuous myofibrils with mitochondria distributed orderly between myofibrils. On the contrary, cardiac tissues from mice of DCM exhibited ruptured and irregular myofibrils, between which mitochondria interspersed disorderly. All these irregular and disordered structures were dramatically improved by THC treatment in the DCM+THC group (Figure 5(c)).

### 3.5. THC Treatment Attenuated Diabetes-Elicited Oxidative Stress by Activating SIRT1 *In Vivo*

**Figure 6:**
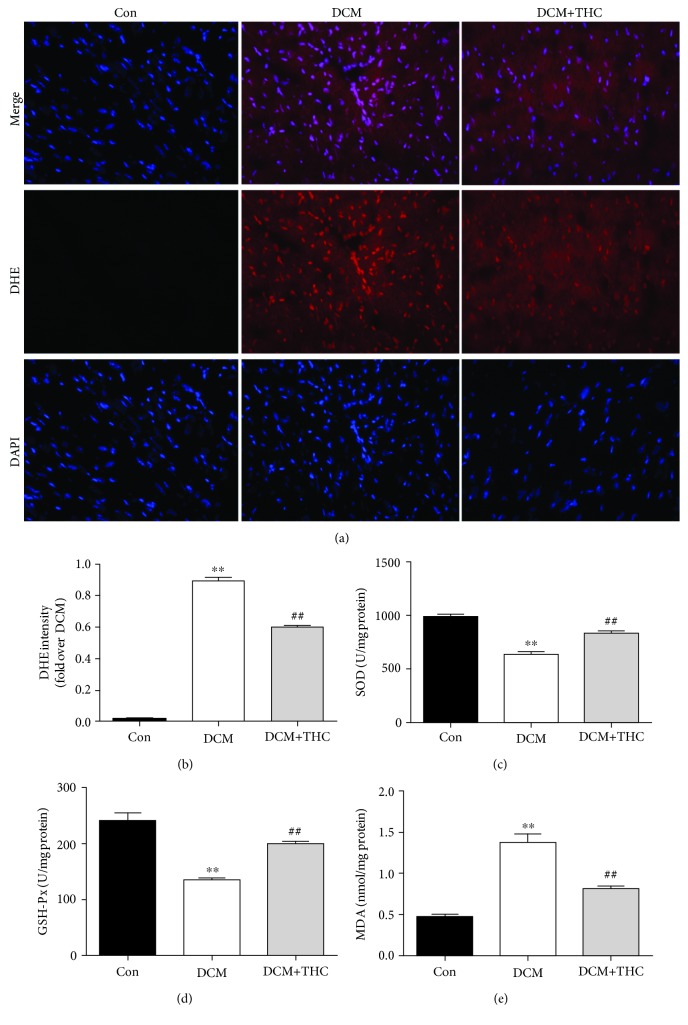
THC treatment alleviated oxidative stress in diabetic cardiomyopathy: (a) representative images of DHE staining (400x); (b) DHE intensity; (c) SOD activity; (d) GSH-Px activity; and (e) MDA contents in fresh myocardial tissues. Data are presented as the mean ± SEM (*n* = 6 in each group). ^∗^^/^^∗∗^*P* < 0.05/0.01 versus the Con group; ^#/##^*P* < 0.05/0.01 versus the DCM group.

In accordance with our results *in vitro*, intracellular ROS production was markedly increased in the DCM mouse heart compared to the Con group, which was determined by the elevated intensity of DHE fluorescence. Consistently, administration with THC for 12 weeks dramatically inhibited the generation of ROS (Figures 6(a) and 6(b)). The antioxidant capacity of THC was further determined by two pivotal enzymes in scavenging oxygen radical, SOD and GSH-Px, together with another oxidative stress indicator lipid peroxide MDA. As shown in Figures 6(c)–6(e), enzymatic activities of SOD and GSH-Px were dramatically decreased accompanied by observably increased MDA content in mouse heart tissues of the DCM group. However, all those changes were significantly reversed by THC treatment. Those results confirmed the protective effectiveness of THC administration to attenuate oxidative stress *in vivo*.

**Figure 7:**
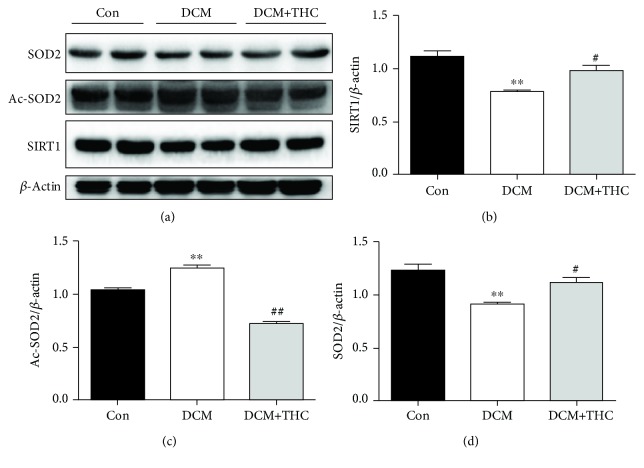
THC treatment upregulated the SIRT1 signaling pathway in diabetic cardiomyopathy. (a) Representative blots of SIRT1, Ac-SOD2, SOD2, and *β*-actin. (b–d) Histogram shows the quantitative expression changes of SIRT1, Ac-SOD2, and SOD2; all of the proteins were normalized to *β*-actin before relative quantitative analysis. Data are presented as the mean ± SEM (*n* = 6 in each group). ^∗^^/^^∗∗^*P* < 0.05/0.01 versus the Con group; ^#/##^*P* < 0.05/0.01 versus the DCM group.

We further detected the protein expression of SIRT1 and its two crucial downstream molecules Ac-SOD2 and SOD2. As Western blot analysis shows in Figures 7(a)–7(d), with the reduced expression of SIRT1, the deacetylated substrate Ac-SOD2 was observably increased; naturally, the deacetylated production SOD2 was accordingly decreased in the DCM group compared to the Con group. Interestingly, treatment of THC dramatically reversed those transformations in the DCM+THC group. Collectively, those data suggest that THC could attenuate diabetes-induced oxidative stress by activating the SIRT1-involved antioxidative pathway in DCM.

### 3.6. THC Treatment Suppressed the ROS-Stimulated TGF*β*1-Smad3 Fibrotic Pathway in Diabetic Hearts

**Figure 8:**
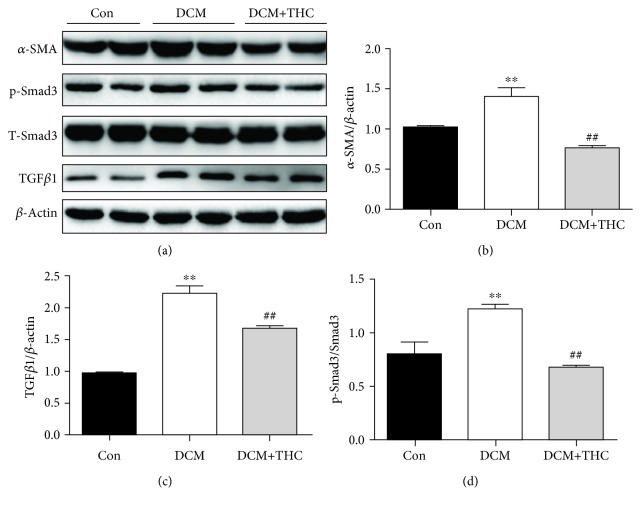
THC treatment restrained stimulation of the TGF*β*1-Smad3 pathway in diabetic cardiomyopathy. (a) Representative blots of TGF*β*1, T-Smad3, p-Smad3, *α*-SMA, and *β*-actin. (b–d) Histogram presents the expression changes of TGF*β*1, p-Smad3, and *α*-SMA among different groups; data are presented as the mean ± SEM (*n* = 6 in each group). ^∗^^/^^∗∗^*P* < 0.05/0.01 versus the Con group; ^#/##^*P* < 0.05/0.01 versus the DCM group.

To further clarify the underlying mechanism connected between the prominent capacities of antioxidative stress and antifibrosis achieved by THC treatment, the TGF*β*1/Smad3 signaling pathway, which plays a key role in mediating ROS generation to pathologic fibrosis and hypertrophy of DCM [12, 28], was further explored in our research. As shown in Figures 8(a)–8(b), the antifibrotic effectiveness of THC was further determined by the protein expression of the cardiac fibrotic marker *α*-SMA, which was dramatically raised in the DCM group and could be significantly inhibited by THC treatment. Furthermore, the TGF*β*1/Smad3 pathway was evidently stimulated in DCM mouse hearts compared to the Con group; consistently, it was also prominently suppressed in the THC-treated DCM group (Figures 8(c) and 8(d)). Those results suggested that inhibition of the TGF*β*1/Smad3 fibrotic signaling pathway was involved in the significant effectiveness of THC to ameliorate fibrosis in diabetic hearts via SIRT1-induced reduction of ROS generation.

## 4. Discussion

Consistent with previous reports, oxidative stress and cardiac fibrosis induced by ROS generation in high-glucose status resulted in myocardial pathological changes and cardiac dysfunctions in DCM. For the first time, our work determined that THC treatment effectively attenuated HG-induced oxidative stress of cardiomyocytes both *in vivo* and *in vitro* and finally prevented cardiac fibrosis and structural disorder and improved myocardial function in DCM mice. Mechanistically, THC-stimulated SIRT1 involved the antioxidative signaling pathway and suppressed TGF*β*1/Smad3-mediated cardiac fibrosis.

ROS generation is overly stimulated by metabolic abnormalities in DCM, such as hyperglycemia, hyperlipidemia, and accumulation of advanced glycation end-products (AGEs), which finally leads to intracellular oxidative stress [3]. Continuously boosted status of oxidative stress has the trend to unduly exhaust endogenous antioxidants, such as SOD and GSH-Px, and accompanied by the accumulation of prooxidative stress products such as MDA in the heart, which are observed both in our study and lots of previous investigations [9, 24, 29]. To resist against the damages from sustained oxidative stress, a series of endogenous antioxidative mechanisms in cells have been evolved, one of which is SIRT1, a histone deacetylase belonging to the family of yeast the silent information regulator 2 (Sir2) [21]. SIRT1 is the first member to be found in this sirtuin family and is also widely studied for its prominent protective effectiveness on cardiovascular diseases [22, 30–33]. Moreover, the multiple benefits for targeting SIRT1 to prevent various angiocardiopathy that involved the status of diabetes were further revealed by numerous finds [31, 34]. Cardellini et al. confirmed the crucial positive effect of increased SIRT1 expression to reduce atherosclerotic plaques in type2 diabetes [35]. Recently, we further determined that the reduced SIRT1 signaling pathway dramatically exacerbated myocardial ischemia/reperfusion injury both in type 1 and type 2 diabetic models, and stimulation of SIRT1 by honokiol or melatonin could significantly ameliorate the damage by preventing oxidative stress and apoptosis [24, 36]. All those evidences suggest that targeting SIRT1 may be a promising treatment strategy for DCM. THC, the major active metabolite of natural product curcumin, possesses a variety of pharmacological activities similar to curcumin, such as antioxidative, anti-inflammatory, anticancer, antidiabetic, and neuroprotective properties [13, 14, 37–40]. Moreover, THC was found to be more stable and shows a higher bioavailability than curcumin, which were generally considered as main limitations for the clinical application of curcumin [37, 41–43]. Further, numerous studies had documented that THC exhibited superior capabilities to reverse abnormalities in diabetic status, such as reduction of blood glucose and lipids, increase of insulin in plasma and the activities of important antioxidative enzymes SOD and GSH-Px, and also attenuation in accumulation of collagen [17, 18, 44–46]. Intriguingly, THC was found to extend the life span of wild-type Drosophila, and the effect was eliminated in the Sir2 null mutants, which meant THC achieve its benefits in a Sir2-dependent way. Although the author further demonstrated that THC presented positive effect on extending the life span of Drosophila in a characteristic similar to resveratrol (RES), which was generally considered as an efficient activator of SIRT1, it is still unknown whether THC could also target SIRT1 as the effector [25]. Thus, we designed this study to test the potential protective effectiveness of THC to mitigate oxidative stress by activating SIRT1 and finally prevent the cardiac pathologic changes of the structure and function in DCM. As expected, the ROS generation exhibited dramatically increased both in the heart tissues from the DCM group and HG-treated H9c2 cells compared with the Con group or cells cultured with LG. Accordingly, the activities of two pivotal antioxidative enzymes, SOD and GSH-Px, were markedly attenuated in diabetic hearts, accompanied with the substantial raised MDA content, a lipid peroxide normally used as an indicator to evaluate the level of oxidative stress. However, with the treatment of THC, all those abnormities were significantly reversed both *in vivo* and *in vitro*, which strongly determined the antioxidative capacity of THC. To further explore whether the protective effectiveness of THC is correlated with SIRT1, the expression of SIRT1 was detected. As our results show, SIRT1 was significantly suppressed under the status of high glucose both in H9c2 cells and mouse hearts of DCM models, which were accordant with previous studies [21, 24, 36]. Interestingly, the expression of SIRT1 was dramatically upregulated by additional administration of THC, which shared the similar trend both *in vivo* and *in vitro*. To further determine that THC achieved the antioxidative effect involved with promoted SIRT1 activities, SOD2, one of the most important deacetylated productions of SIRT1, which plays a pivotal role in scavenging ROS generation, and the correspondent deacetylated substrate Ac-SOD2 were monitored in our studies. SOD2, also called as manganese superoxide dismutase, is the major enzymatic superoxide scavenger localized in the mitochondria [47]. As mitochondrial dysfunction caused by metabolic disorders is the main origin of ROS generation in DCM [10, 48], SOD2 has an extremely pivotal role in regulating ROS homeostasis after being deacetylated from Ac-SOD2 by the effect of several kinds of sirtuins, in which SIRT1 plays a crucial role [47, 49]. It was reported that adenovirus-mediated overexpression of SIRT1 significantly reversed the decreased expression of SOD2 induced by high-glucose conditions in human umbilical vein endothelial cells [34]. However, whether THC could prevent oxidative stress in DCM by promoting the SIRT1-deacetylated SOD2 is still unknown. In our studies, significant reduction of deacetylated SOD2 accompanied by remarkable increasing deacetylated substrate Ac-SOD2 was observed both in HG-induced H9c2 cells and diabetic hearts from the DCM group with the suppression of SIRT1. Moreover, treatment of THC dramatically reversed those transformations by increasing the expression of SIRT1 and deacetylated SOD2 followed by reduced deacetylated substrate Ac-SOD2. Collectively, all those data further suggested that THC could attenuate high glucose-induced oxidative stress by activating SIRT1 and the increasing deacetylation on SOD2 both *in vivo* and *in vitro*.

Myocardial fibrosis exists as one of the main pathological structural changes in both type I and type II diabetes [50]. It is reported that the collagen level was increased in left ventricular endomyocardial biopsies from diabetes patients without coronary disease, which was accompanied with reduced LVEF [51]. In our study, severe cardiac fibrosis was also observed in DCM groups, which was further demonstrated by the accumulation of collagen I/III and increased expression of *α*-SMA in the heart. Increased ROS generation induced by chronic stimulation from high glucose was suggested to be closely related to the severity and pathogeny of diabetes-induced cardiac fibrosis [52]. The TGF*β*1/Smad3 pathway has been proved to be the most important signaling pathway in mediating the ROS-induced process of cardiac fibrosis [9, 12, 28]. It was determined that significantly decreased cardiac fibrosis and increased myocardial compliance were observed in the transgenic mouse with Smad3 deficiency [12]. In our study, we demonstrated that the remarkable effectiveness to reduce cardiac fibrosis in DCM by administration of THC was achieved, at least partially, by the inhibition of the TGF*β*1/Smad3 profibrotic signaling pathway via SIRT1-induced attenuation of ROS generation. Of course, as we mentioned before, the effect of deacetylation was often accomplished by the collaboration of several kinds of sirtuins. Zhai et al. in our group found that SIRT1 mediated the protective effect of melatonin to prevent myocardial ischemia/reperfusion injury by increasing deacetylation on SOD2 with the dependent assistance from SIRT3, another member in the sirtuin family [47]. Intriguingly, SIRT4, another member of this family, was reported to play a negative role in the deacetylated progress of SOD2 by inhibiting the binding of SOD2 to SIRT3, which finally increased ROS generation and aggravation of cardiac fibrosis and dysfunction in Ang II-induced pathological cardiac hypertrophy [53]. Therefore, future investigations will still need to answer, whether there are other sirtuins taking part in the protectively antioxidative and antifibrotic effects of THC treatment in DCM via increasing SIRT1 and deacetylated SOD2 and the further interrelationship between those sirtuins.

## 5. Conclusion

In this study, we have demonstrated that THC treatment significantly increased the expression of SIRT1 and deacetylated SOD2 both *in vitro* and *in vivo*, thus prevented cardiomyocytes against oxidative damage and inhibited the ROS-induced TGF*β*1/Smad3 profibrotic signaling pathway, which finally alleviated myocardial hypertrophy and cardiac dysfunction in DCM. Our findings confirmed the potential for using THC as a promising therapeutic or adjuvant drug in the treatment of DCM. Moreover, we further determined the crucial roles of oxidative stress and ROS-induced fibrosis in the pathological process of DCM, and SIRT1 may be a vital effector molecule for natural products, such as THC, to target oxidative stress and fibrosis induced by ROS generation. In the future, the synergic relationship between SIRT1 and other sirtuins from the Sir2 family in the protectively increasing deacetylation on SOD2 by THC administration will be further explored and elucidated in our studies.

## Data Availability

The data used to support the findings of this study are available from the corresponding author upon request.
